# Small Mammal Investigation in Spotted Fever Focus with DNA-Barcoding and Taxonomic Implications on Rodents Species from Hainan of China

**DOI:** 10.1371/journal.pone.0043479

**Published:** 2012-08-29

**Authors:** Liang Lu, Douglas Chesters, Wen Zhang, Guichang Li, Ying Ma, Huailei Ma, Xiuping Song, Haixia Wu, Fengxia Meng, Chaodong Zhu, Qiyong Liu

**Affiliations:** 1 Department of Vector Biology and Control, National Institute for Communicable Disease Control and Prevention, Chinese Center for Disease Control and Prevention, Beijing, China; 2 Department of Bioinformatics, National Institute for Communicable Disease Control and Prevention, Chinese Center for Disease Control and Prevention, Beijing, China; 3 State Key Laboratory for Infectious Diseases Prevention and Control, Beijing, China; 4 Institute of Zoology, Chinese Academy of Sciences, Beijing, China; 5 QingHai Institute for Endemic Disease Prevention and Control, Xining, Qinghai, China; University of Vermont, United States of America

## Abstract

Although mammals are a well-studied group of animals, making accurate field identification of small mammals is still complex because of morphological variation across developmental stages, color variation of pelages, and often damaged osteological and dental characteristics. In 2008, small mammals were collected for an epidemiological study of a spotted fever outbreak in Hainan, China. Ten species of small mammals were identified by morphological characters in the field, most using pelage color characters only. The study is extended here, in order to assess whether DNA barcoding would be suitable as an identification tool in these small mammals. Barcode clusters showed some incongruence with morphospecies, especially for some species of *Rattus* and *Niviventer*, so molecular delineation was carried out with an expanded dataset of combined cytochrome b (Cyt-*b*) and cytochrome c oxidase subunit I (COI) sequences. COI sequences were successfully amplified from 83% of collected mammals, but failed in all specimens of *Suncus murinus*, which were thus excluded in DNA barcoding analysis. Of note, ten molecular taxonomic units were found from samples of nine morphologically identified species. Accordingly, 11 species of small mammals were present in the investigated areas, including four *Rattus* species, three *Niviventer* species, *Callosciurus erythraeus*, *Neohylomys hainanensis*, *Tupaia belangeri*, and *Suncus murinus*. Based on the results of the phylogenetic and molecular delineation analyses, the systematic status of some rodent species should be redefined. *R. rattus hainanicus* and *R. rattus sladeni* are synonyms of *R. andamanensis*. *R. losea* from China and Southeast Asia comprises two independent species: *R. losea* and *R. sakeratensis*. Finally, the taxonomic status of three putative species of *Niviventer* should be further confirmed according to morphological, molecular and ecological characters.

## Introduction

Rodents are important host animals for many zoonoses that threaten public health worldwide [1], [2]. Typically, there is a specific association between pathogens and host animals, such as the coevolutionary relationship between hantavirus and their rodent hosts [3]. Thus, gaining accurate taxonomic information on host animals is important for surveillance and epidemiological investigation of rodent-borne diseases.

Mammals rank amongst the most studied animal groups, with their taxonomy and species diversity well documented in the literature [4]. However, field identification of many small mammal species remains difficult, in large part because of morphological variation through development, and color variation of pelages (mammalian coat) between individuals. Only through analysis of internal morphology (e.g. skull and dentition) can definitive identification be made. Furthermore, molecular data from one previous study suggests the frequent occurrence of cryptic mammal species that are overlooked when using morphological characters alone [5]. Therefore, a standard molecular identification system is necessary as a complement to morphological methods, in order to reduce uncertainties in the identification of mammal species.

One standardized molecular identification approach, termed DNA barcoding [6], [7], has been extensively used in recent years. This technique can also provide genetic references to validate field identifications made by researchers with limited taxonomic background, which makes it a particularly valuable tool for conducting ecological and epidemiological surveys. Previous applications of this technique in primates and small mammals indicate that it is a valuable method for species identification [8], [9], [10], and DNA barcoding has been instrumental in reassessing the species diversity of regional faunas of small mammals and other taxa [11], [12], [13].

Three potential outcomes of using DNA barcodes in the investigation of species diversity of a specific geographic region are: 1) morphologically homogeneous specimens sharing DNA barcodes with little intraspecific variation; 2) morphologically homogeneous specimens possessing DNA barcodes divergent at a level beyond that expected for species, indicating the possibility of overlooked species [9], [10], [14]; 3) a putative new species or a new record species for the area [9]. When a cryptic species or new species are indicated, a study of their systematic position becomes necessary. However, because systematic information content of the COI barcode is limited, this fragment alone is insufficient for reliable molecular phylogenetic reconstruction and the assignment of new species [15]. Therefore, species delimitation approaches based on multi-locus phylogenies are necessary to define the species status of studied samples, and clarify the relationship with other closely related species. However, using certain phylogenetic based species delimitation methods [16], [17], [18], subjective judgment regarding morphologic and ecological traits are necessary in order to determine whether a highly supported clade should be considered an independent species. Pons et al. [19] proposed a statistical method of DNA-based species delimitation which determines the switch or threshold point of transition from species-level to population-level branching on a phylogenetic tree, giving an estimate of the number of species. This method has been successfully used in species delimitation of asexual mites [20] and rodents of the Rattini tribe from Southeast Asia [21].

In 2007, a severe spotted fever case was reported in Hainan, China [22], which prompted epidemiological investigation. The investigation was carried out in three different counties in the north and central areas of the province, and focused on reservoir animals carrying the *Rickettsia* bacteria that are responsible for spotted fever. Investigators collected blood and/or tissue samples of livestock, pets, and small mammals (rodents, moonrats, and shrews) for the study. However, identification of the small mammals, carried out prior to collection of blood and tissue samples, was hasty and by means of external morphological characteristics only. Confirmation of the species assignments is necessary to ensure the utility of the epidemiology studies.

Although it is a well-studied group of rodents, there are still some disagreements about species classification in *Rattus*, such as *R. rattus* occupying China, and *R. losea*. There are four subspecies of *R. rattus* recorded in China: namely *R. rattus rattus*, *R. rattus alexandrinus*, *R. rattus sladeni* and *R. rattus hainanicus* [23]. Among them, *R. rattus rattus* and *R. rattus alexandrinus* were regarded as imported subspecies, while *R. rattus sladeni* and *R. rattus hainanicus* were native to southern mainland China, and Hainan Island, respectively [23]. However, Musser and Carleton [24] downgraded *R. rattus sladeni* to a synonym of *R. tanezumi*, and downgraded *R. rattus hainanicus* to a synonym of *R. andamanensis*. *R. losea* was described from Taiwan, and recorded in China and other countries of Southeast Asia [24]. This species is discontinuously distributed across mainland Southeast Asia and East Asia in general, and displays two regional forms in its morphology [25] and genetics [26]. Pagès et al. [21] studied the taxonomy of the Rattini tribe based on samples collected from Southeast Asia, using a phylogenetic-based species delimitation method. The authors were hesitant to name one putative species as *R. losea* because only specimens of Southeast Asia were included, although these specimens were all morphologically identified as *R. losea*. Aplin et al. [27] confirmed subsequently that *R. losea* from Taiwan and Southeast Asia were two independent species, and named the latter *R. sakeratensis*.

The genus *Niviventer* occurs in China and Southeast Asia, with 17 species recorded [24]. The characters traditionally used to distinguish these species are not completely unequivocal, especially amongst some closely related species. Musser examined a large number of specimens to give morphological and geographic species-limits to some morphologically homogeneous species [28], [29], [30]. However, identification remains difficult even from areas with detailed species records, for example, two putative species reported by Pagès et al. [21]. Jing et al. studied the molecular phylogeny of *Niviventer* species of China [31], but their species identification was later questioned [21]. The karyotype studies of different *Niviventer* species summarized by Li et al. [32] also suggested that there are identification problems in these taxa.

Therefore, in the present study, we use DNA barcoding to confirm the field identification assignments, and highlight rodent species prone to misidentification. After barcoding identification, we investigate the taxonomic status of some popular rodent species in Hainan further, including species of *Rattus* and *Niviventer*, using molecular species delimitation.

## Materials and Methods

### Ethical statement

Ethical approval for this study was obtained from the Ethical Committee of Chinese Center for Disease Control and Prevention. Small mammals were live trapped in areas which were not privately owned or protected. All small mammals involved in this paper were neither endangered nor listed as protected species. No specific permits were required for the described field studies.

### Animal collection

**Figure 1:**
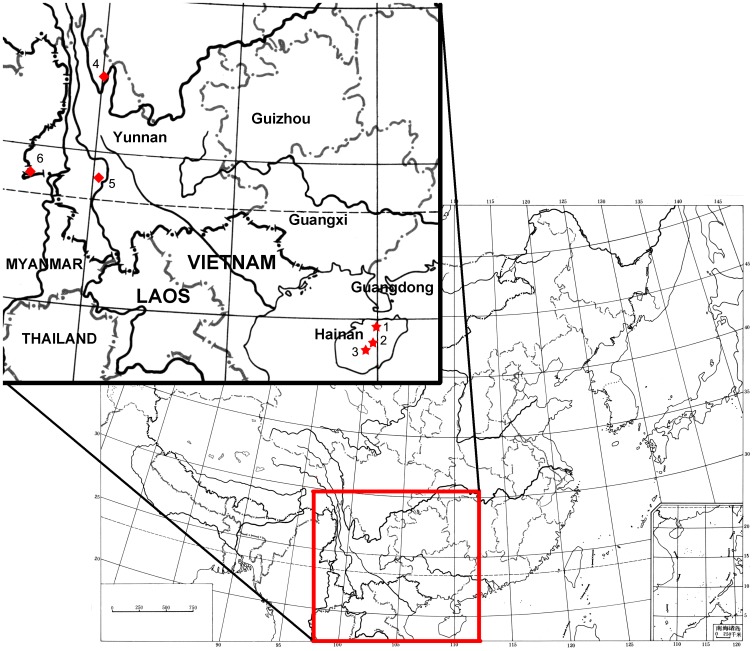
Sample locations of the small mammal collected in Hainan, and *Rattus rattus sladeni* caught from Yunnan. 1: Chengmai, 2: Qiongzhong, 3: Wuzhishan, 4: Deqin, 5: Lincang and 6: Ruili.

Host animals were collected from three counties of Hainan, including Chengmai, Qiongzhong, and Wuzhishan. Small mammals were live trapped indoors, on farmlands and forests, around villages in these counties (Figure 1). Trapped small mammals were euthanized with CO_2_ before species identification and sample collection. After standard morphological identification (based on color of pelage, and length of body and tail) [23], blood, liver and spleen samples were collected, stored in liquid nitrogen in the field, and maintained at −80°C in the laboratory until used for experiments. Some material from specimens in good condition were kept as voucher specimens, including skull and pelages. All voucher specimens were identified by taxonomic experts after the field work. Field sample identifications and locality information are listed in Table 1.

**Table 1:** Information of small mammals collected from Hainan, including field identification results, number of individuals, collection site, and accession numbers of COI and Cyt-*b* sequences submitted to Genbank.

Field identification	Number of individuals	Locality	Voucher specimens	COI	Cyt-*b*
*Rattus losea*	28	Qiongzhong		HM031871–HM031896	HM031709–HM031721
	5	Chengmai			
*Rattus rattus hainanicus*	5	Qiongzhong	HN115	HM031790–HM031836	HM031722–HM031763
	6	Wuzhishan			
	42	Chengmai			
*Rattus norvegicus*	2	Qiongzhong		HM031897–HM031910	HM031676–HM031682
	17	Chengmai			
*Rattus tanezumi*	33	Chengmai		HM031837–HM031870	HM031683–HM031708
	1	Qiongzhong			
*Niviventer confucianus*	3	Qiongzhong	HN113	HM031911–HM031913	JF714939, JF714941
	1	Wuzhishan			
	4	Chengmai			
*Niviventer fulvescens*	6	Wuzhishan	HN131, HN162	HM031914–HM031931	HM031673–HM031675
	13	Chengmai			JF714932–JF714938
	3	Qiongzhong			JF 714940, JF714942
*Callosciurus erythraeus*	4	Qiongzhong	HN120	HM031932–HM031935	N/A
*Suncus murinus*	4	Wuzhishan	–	N/A	N/A
*Neohylomys hainanensis*	2	Qiongzhong	HN122	HM031764–HM031766	N/A
	1	Wuzhishan			
*Tupaia belangeri*	25	Chengmai	–	HM031767–HM031789	N/A

### Sequence acquisition

Total genomic DNA of small mammals was isolated using the Qiagen DNAeasy blood and tissue DNA isolation kit (Qiagen China, Pudong, Shanghai) according to the manufacturer's instructions. To amplify 650 bp of the cytochrome c oxidase subunit 1 gene (COI), universal primers BatL5310, R6036R and related amplification conditions were used according to Robins et al. [33]. If amplification with the universal primers failed, the cocktail primer sets were used instead [34]. Although the cocktail primer set was initially designed for fish DNA barcoding, it was successfully used in barcoding of bats [9], and pikas and shrews in author's laboratory. Conditions for the cocktail primer sets were: 94°C for 1 min, five cycles of 94°C for 30 s, 50°C for 40 s, and 72°C for 1 min, followed by 35 cycles of 94°C for 30 s, 54°C for 40 s, and 72°C for 1 min, with a final extension at 72°C for 10 min. For samples from species of *Rattus* and *Niviventer*, 1200 bp of the mitochondrial cytochrome b gene (Cyt-*b*) were also amplified using the primers L14724 and H15915 of Irwin et al. [35]. Each PCR cycle consisted of 93°C for 1 min, 50°C for 1 min and 72°C for 2 min. The cycle was repeated 35 times with a final extension at 72°C for 10 min. All amplicons were directly sequenced in both directions with the ABi 3100 automatic sequencer (Perkin–Elmer, Waltham, MA) using the ABi PRISM BigDye Terminator Cycle Sequencing Ready Reaction Kit with AmpliTaq DNA polymerase (Applied Biosystems, Foster City, CA). COI and Cyt-*b* sequences of *Rattus* and *Niviventer* obtained by Pagès et al. [21] showed substantial overlap with the fragments used here, and so were downloaded from GenBank and added to the dataset. COI and Cyt-*b* sequences of *R. rattus sladeni* from Yunnan (Figure 1) were also included in the dataset to confirm the status of the subspecies. Information of sequences of Pagès et al. [21] and *R. rattus sladeni* were listed in Table 2.

**Table 2:** Information of sequences from previous studies and *R. rattus sladeni* included in the dataset.

Sample number	Locality	Field Identification	Phylogenetic species*	Reference papers	COI	Cyt b
R2953	Kanchanaburi (Thailand)	*Rattus tanezumi*	*Rattus andamanensis*	[21]	HM217525	HM217396
R3087	Kanchanaburi (Thailand)	*Rattus andamanensis*	*Rattus andamanensis*	[21]	HM217533	HM217403
CB0001	Veal Renh (Cambodia)	*Rattus argentiventer*	*Rattus argentiventer*	[21]	HM217484	HM217362
CB0104	Veal Renh (Cambodia)	*Rattus argentiventer*	*Rattus argentiventer*	[21]	HM217486	HM217364
R0284	Ratchaburi (Thailand)	*Rattus exulans*	*Rattus exulans*	[21]	HM217508	HM217377
R0856	Nakhon Pathom (Thailand)	*Bandicota indica*	*Rattus exulans*	[21]	HM217510	HM217379
R2795	Ratchaburi (Thailand)	*Rattus exulans*	*Rattus exulans*	[21]	HM217527	HM217395
R3520	Sakhon Nakhon (Thailand)	*Rattus exulans*	*Rattus exulans*	[21]	HM217553	HM217424
R4004	Kalasin (Thailand)	*Rattus exulans*	*Rattus exulans*	[21]	HM217564	HM217437
R5349	Nan (Thailand)	*Rattus exulans*	*Rattus exulans*	[21]	HM217595	HM217470
R5447	Nan (Thailand)	*Rattus exulans*	*Rattus exulans*	[21]	HM217596	HM217472
L0010	Luang Prabang (LPDR)	*Rattus sp.*	*Rattus nitidus*	[21]	HM217488	HM217474
L0180	Luang Prabang (LPDR)	*Rattus nitidus*	*Rattus nitidus*	[21]	HM217492	HM217478
L0192	Luang Prabang (LPDR)	*Rattus nitidus*	*Rattus nitidus*	[21]	HM217493	HM217479
R0115	Ratchaburi (Thailand)	*Rattus norvegicus*	*Rattus norvegicus*	[21]	HM217501	HM217370
R0223	Ratchaburi (Thailand)	*Rattus norvegicus*	*Rattus norvegicus*	[21]	HM217504	HM217373
MDZ10Mada	Madagascar	*Rattus rattus*	*Rattus rattus*	[21]	hM217495	HM217368
ratcosR12	Oman	*Rattus rattus*	*Rattus rattus*	[21]	HM217496	HM217366
ratcosT820	India	*Rattus rattus*	*Rattus rattus*	[21]	HM217498	HM217367
ratcosTE4264	Tanzania	*Rattus rattus*	*Rattus rattus*	[21]	HM217497	HM217365
R0237	Ratchaburi (Thailand)	*Rattus losea*	*Rattus sakeratensis*	[21], [27]	HM217505	HM217374
R0238	Ratchaburi (Thailand)	*Rattus losea*	*Rattus sakeratensis*	[21], [27]	HM217506	HM217375
R1015	Nakhon Ratchasima (Thailand)	*Rattus losea*	*Rattus sakeratensis*	[21], [27]	HM217512	HM217381
R3484	Loei (Thailand)	*Rattus losea*	*Rattus sakeratensis*	[21], [27]	HM217550	HM217421
R3510	Phrae (Thailand)	*Rattus losea*	*Rattus sakeratensis*	[21], [27]	HM217552	HM217423
R4203	Phrae (Thailand)	*Rattus losea*	*Rattus sakeratensis*	[21], [27]	HM217570	HM217443
R4230	Loei (Thailand)	*Rattus losea*	*Rattus sakeratensis*	[21], [27]	HM217573	HM217446
R4402	Loei (Thailand)	*Rattus losea*	*Rattus sakeratensis*	[21], [27]	HM217581	HM217454
CB0028	Veal Renh (Cambodia)	*Rattus tanezumi*	*Rattus sp.*	[21]	HM217485	HM217363
R0169	Ratchaburi (Thailand)	*Rattus tanezumi*	*Rattus sp.*	[21]	HM217503	HM217372
R1818	Prachinburi (Thailand)	*Rattus tanezumi*	*Rattus sp.*	[21]	HM217520	HM217389
R2976	Nakhon Pathom (Thailand)	*Rattus andamanensis*	*Rattus sp.*	[21]	HM217528	HM217397
R3029	Bangkok (Thailand)	*Rattus tanezumi*	*Rattus sp.*	[21]	HM217530	HM217399
R4188	Phrae (Thailand)	*Rattus sp.*	*Rattus sp.*	[21]	HM217569	HM217442
L0100	Luang Prabang (LPDR)	*Rattus tanezumi*	*Rattus tanezumi*	[21]	HM217489	HM217475
L0194	Luang Prabang (LPDR)	*Rattus tanezumi*	*Rattus tanezumi*	[21]	HM217494	HM217480
R3122	Kanchanaburi (Thailand)	*Rattus tanezumi*	*Rattus tanezumi*	[21]	HM217537	HM217407
R3214	Kanchanaburi (Thailand)	*Rattus tanezumi*	*Rattus tanezumi*	[21]	HM217540	HM217410
R3548	Phrae (Thailand)	*Rattus andamanensis*	*Rattus tanezumi*	[21]	HM217555	HM217426
R3573	Nakhon Pathom (Thailand)	*Rattus tanezumi*	*Rattus tanezumi*	[21]	HM217558	HM217430
R4003	Kalasin (Thailand)	*Rattus tanezumi*	*Rattus tanezumi*	[21]	HM217563	HM217436
R4377	Loei (Thailand)	*Rattus andamanensis*	*Rattus tanezumi*	[21]	HM217579	HM217452
R4424	Phrae (Thailand)	*Rattus tanezumi*	*Rattus tanezumi*	[21]	HM217582	HM217456
R4436	Phrae (Thailand)	*Rattus tanezumi*	*Rattus tanezumi*	[21]	HM217583	HM217457
R4481	Phrae (Thailand)	*Rattus andamanensis*	*Rattus tanezumi*	[21]	HM217584	HM217458
R5294	Nan (Thailand)	*Rattus tanezumi*	*Rattus tanezumi*	[21]	HM217592	HM217466
R5296	Nan (Thailand)	*Rattus tanezumi*	*Rattus tanezumi*	[21]	HM217593	HM217467
R1833	Nakhon Sri Thammarat (Thailand)	*Rattus tanezumi*	*Rattus tiomanicus*	[21]	HM217522	HM217391
R3427	Kanchanaburi (Thailand)	*Niviventer sp.*	*Niviventer fulvescens*	[21]	HM217545	HM217416
R3429	Loei (Thailand)	*Niviventer sp.*	*Niviventer fulvescens*	[21]	HM217546	HM217417
R3459	Loei (Thailand)	*Niviventer sp.*	*Niviventer fulvescens*	[21]	HM217548	HM217419
R4525	Loei (Thailand)	*Niviventer sp.*	*Niviventer fulvescens*	[21]	HM217589	HM217464
R4723	Loei (Thailand)	*Niviventer fulvescens*	*Niviventer fulvescens*	[21]	HM217591	HM217465
R4497	Phrae (Thailand)	*Niviventer sp.*	*Niviventer fulvescens.*	[21]	HM217587	HM217461
R3795	Khammouane (LPDR)	*–*	*Niviventer langbianis*	[21]	HM217561	HM217433
R3796	Khammouane (LPDR)	*–*	*Niviventer langbianis*	[21]	HM217562	HM217434
R3212	Kanchanaburi (Thailand)	*Niviventer langbianis*	*Niviventer sp*1.	[21]	HM217539	HM217409
LC104	Lincang, Yunnan (China)	*Rattus rattus sladeni*	*Rattus andamanensis*	This paper	JQ793910	JQ793904
LC135	Lincang, Yunnan (China)	*Rattus rattus sladeni*	*Rattus andamanensis*	This paper	JQ793914	JQ793902
LC136	Lincang, Yunnan (China)	*Rattus rattus sladeni*	*Rattus andamanensis*	This paper	JQ793912	JQ793906
LC140	Lincang, Yunnan (China)	*Rattus rattus sladeni*	*Rattus andamanensis*	This paper	JQ793913	JQ793903
RL038	Ruili, Yunnan (China)	*Rattus rattus sladeni*	*Rattus andamanensis*	This paper	JQ793916	JQ793908
RL039	Ruili, Yunnan (China)	*Rattus rattus sladeni*	*Rattus andamanensis*	This paper	JQ793917	JQ793909
RL055	Ruili, Yunnan (China)	*Rattus rattus sladeni*	*Rattus andamanensis*	This paper	JQ793911	JQ793905
DQ366	Deqin, Yunnan (China)	*Rattus rattus sladeni*	*Rattus andamanensis*	This paper	JQ793920	JQ793901
DQ372	Deqin, Yunnan (China)	*Rattus rattus sladeni*	*Rattus andamanensis*	This paper	JQ793918	JQ793899
DQ373	Deqin, Yunnan (China)	*Rattus rattus sladeni*	*Rattus andamanensis*	This paper	JQ793919	JQ793900

### Phylogenetic analyses

All sequences were aligned using CLUSTALW [36] and manually confirmed. The COI and Cyt-*b* gene sequences of specimens of *Rattus* and *Niviventer* were aligned separately, and trimmed to a common length before concatenation. Neighbour Joining (NJ) trees based on COI sequences were generated using K2P distances, calculated in Paup*4b [37]. Missing data were ignored for distance calculation, and ties were broken at random. Phylogenies were generated from the complete dataset using Maximum likelihood (ML) and Bayesian inference (BI) approaches. *Micromys minutus* (HM217360, HM217482) was selected as the outgroup in all analyses. For use in model based tree inferences, the best fit substitution models were determined for the two partitions (COI and Cyt-*b*) using Likelihood ratio tests [38], [39] implemented in Jmodeltest0.1 [40]. The TPM1uf+G model was selected for COI of *Rattus* and *Niviventer* species, and the TIM2+I+G model was selected for Cyt-*b* sequences. ML trees were inferred using Garli v2.0 [41], a software allowing the implementation of partitioned evolutionary models. The best fit model for each gene was input via the starting model option (the ‘streefname’ option given in the configuration file), and these values fixed. Then a partitioned search was performed with otherwise default settings. Node support was obtained via bootstrapping, with the topology termination threshold (parameter: genthreshfortopoterm) reduced to 1000 to increase search speed. Bayesian trees were inferred using MrBayes v3.1.2 [42], again with a partitioned model. The Bayesian search was run for 2 million generations, sampling every 500, with two independent runs performed, each consisting of three heated and one cold chain. Convergence was assessed using the standard deviation of split frequencies, and the estimated sample sizes (ESS) of the sampled parameters, as calculated using Tracer [43].

Molecular delineation was carried out on a dataset from which identical haplotypes were removed (according to the algorithm given by [44]). The dereplicated dataset consisted of 52 sequences, with a total length of 1789 bp. A NJ tree was first generated, using Paup*4b [37]. Genetic distances were calculated under the K2P model, where missing data were ignored in distance calculation, and ties broken at random. ML and BI trees were also inferred for the dereplicated dataset, using the same method as used for the analysis of the complete dataset. The phylogenies from the three different methods were clock constrained using r8s 1.71 [45]. The root node was fixed at an arbitrary value of 1.0, then ultrametric trees formed by penalized likelihood (PL) and non-parametric rate smoothing (NPRS). For PL, smoothing parameters were compared by cross calibration (r8s command: divtime method = pl crossv = yes cvstart = −3 cvinc = 1 cvnum = 9), with the optimal value (10), used in further analyses. Finally, the putative species units on the ultrametric trees were determined using the general mixed Yule coalescent (GMYC) method [19]. This procedure detects the switch in the rate of lineage branching in a tree, from interspecific long branches to intraspecific short branching, and identifies clusters of specimens corresponding to putative species. A threshold (T) is optimized with the GMYC model so that nodes before the threshold are considered as species diversification events, therefore the number of species can be estimated. Significance was assessed by likelihood ratio test against a null model of a single coalescent population. This test was implemented using R code provided by T. G. Barraclough.

## Results

A total of 205 small mammals were collected from three counties of Hainan. According to the morphological criteria we used, seven species belonged to three genera of Rodentia, two species belonged to two genera of Soricomorpha, and there was one species of Scandentia (Table 1).

**Figure 2:**
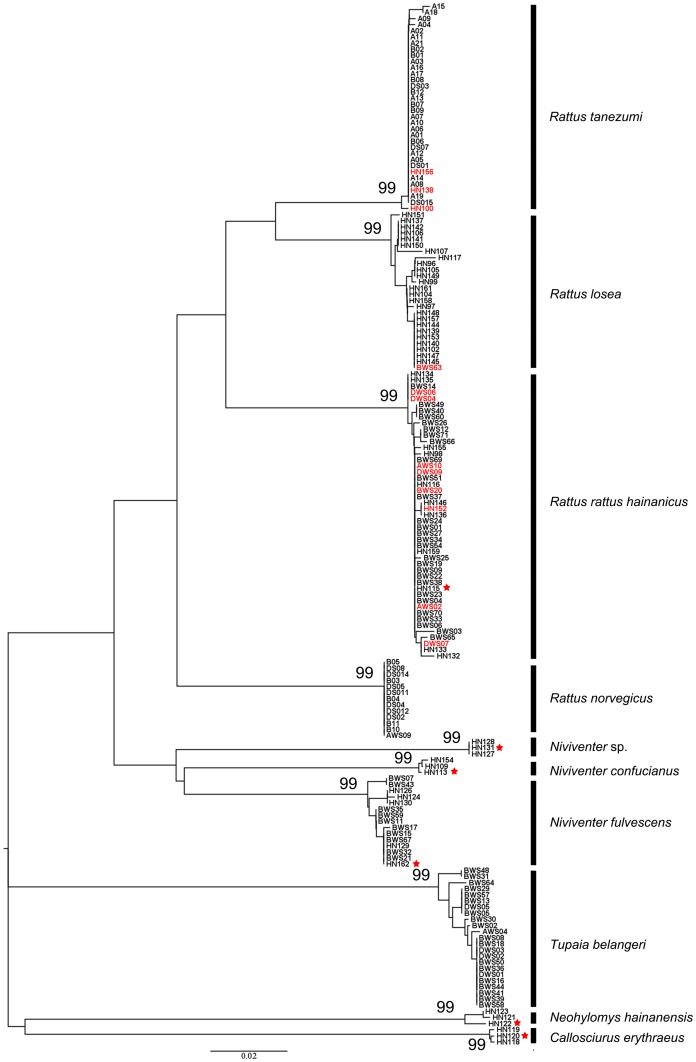
Neighbor-joining tree of COI sequence for small mammals collected from Hainan. Samples separated by small genetic distance (<2%) were labeled with one vertical black bar and regarded as one species. The red sample names meant they were misidentified in the field. The samples labeled with red star indicated the availability of voucher specimens.

COI amplicons, each approximately 650 bp in length, were recovered from 172 individual animals (83%). Of note, amplification failed in all specimens of *Suncus murinus*, even with the cocktail primer set. The NJ tree of COI sequence from Hainan showed that there were ten well supported lineages (Figure 2). Nine of these lineages corresponded to field identified species, but three specimens identified (via morphology) as *N. confucianus* were not clustered with other members of this species in the tree. These three specimens (one cluster with two members, and a singleton) are labeled as *Niviventer* sp in Figure 2. According to the NJ tree, 12 specimens were clustered in a lineages different to that given by the field identification, indicating field misidentification (Table 3). These specimens were identified as species of *Rattus*, of which juveniles were particularly difficult to be distinguished.

**Table 3:** Information of samples misidentified in the field of Hainan.

Sample number	Locality	Field identification	Barcoding identification	GenBank accession numbers of COI
HN100	Qiongzhong	*Rattus norvegicus*	*Rattus tanezumi*	HM031870
HN138	Qiongzhong	*Rattus losea*	*Rattus tanezumi*	HM031864
HN152	Qiongzhong	*Rattus losea*	*Rattus rattus hainanicus*	HM031818
HN156	Qiongzhong	*Rattus losea*	*Rattus tanezumi*	HM031865
AWS02	Chengmai	*Niviventer fulvescens*	*Rattus rattus hainanicus*	HM031797
AWS10	Chengmai	*Niviventer fulvescens*	*Rattus rattus hainanicus*	HM031796
BWS20	Chengmai	*Rattus losea*	*Rattus rattus hainanicus*	HM031823
BWS63	Chengmai	*Rattus rattus hainanicus*	*Rattus losea*	HM031872
DWS04	Chengmai	*Rattus losea*	*Rattus rattus hainanicus*	HM031806
DWS06	Chengmai	*Rattus losea*	*Rattus rattus hainanicus*	HM031807
DWS07	Chengmai	*Rattus losea*	*Rattus rattus hainanicus*	HM031790
DWS09	Chengmai	*Rattus losea*	*Rattus rattus hainanicus*	HM031813

The average K2P distances between individuals of *R. norvegicus* was 0, the distance between individuals of *R. tanezumi* 0.08%, and that of *R. rattus hainanicus* and *R. losea* was 0.21% and 0.41% respectively. The average intraspecific distance among different *Niviventer* species ranged from 0 to 0.40%, similar with that of *Rattus* species. We determined that the divergences between *Rattus* species ranged from 7% to 13%, and 11%–14% for *Niviventer* species.

**Figure 3:**
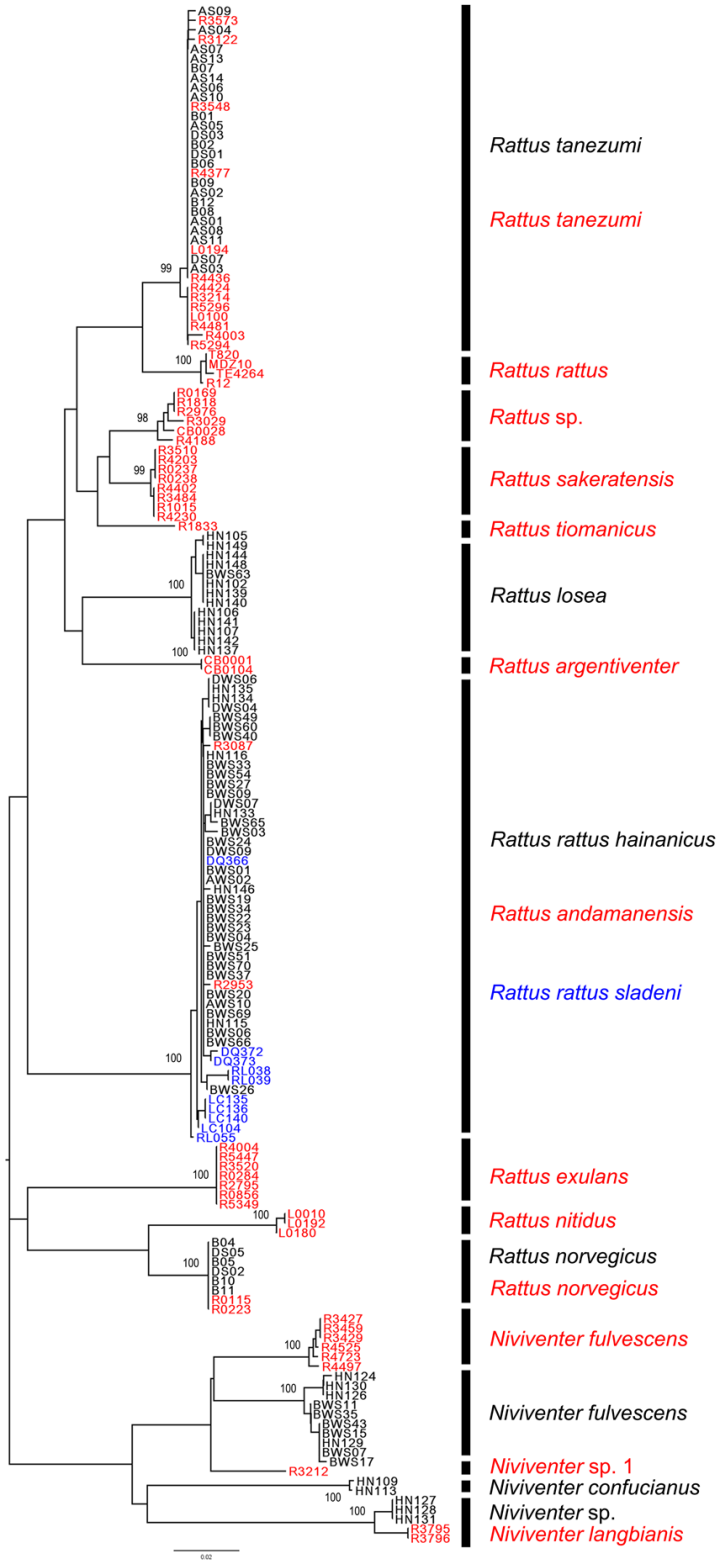
Neighbor-joining tree of COI sequence of *Rattus* and *Niviventer* collected from Hainan, Yunnan and Southeast Asia. Samples with black name and species name were collected from Hainan, those with blue name and species name were from Yunnan, and the red from Southeast Asia [21]. Samples separated by small genetic distance (<2%) were labeled with one vertical black bar and regarded as one species. Low bootstrap support value (<90%) on deep branches were not shown.

The NJ tree of the combined COI dataset from Hainan and Southeast Asia (Figure 3) showed that *R. tanezumi* and *R. norvegicus* of Hainan clustered with samples of the same species from Southeast Asia, *R. losea* of Hainan formed an independent cluster from the *R. sakeratensis* (*R. losea*-like in Pagès et al. [21]) collected from Southeast Asia, and *R. rattus hainanicus*, and *R. rattus sladeni* from Yunnan grouped with *R. andamanensis* from Southeast Asia. The average intraspecific distance of *Rattus* species was 0.23%, ranging from 0 (*R. norvegicus*) to 1.30% (*R. andamanensis*). The interspecific distance of *Rattus* species ranged from 5.5% to 15%. For *Niviventer* species, *N. fulvescens* of Hainan clustered independently from *N. fulvescens* of Southeast Asia, and two samples of Hainan grouped with two samples of *N. langbianis* from Laos. The average intraspecies distance of *Niviventer* species ranged from 0.20% (*N. confucianus*) to 1.60% (*N. langbianis*). The interspecies distance of *Niviventer* species ranged from 14.0%–18.9%.

**Figure 4:**
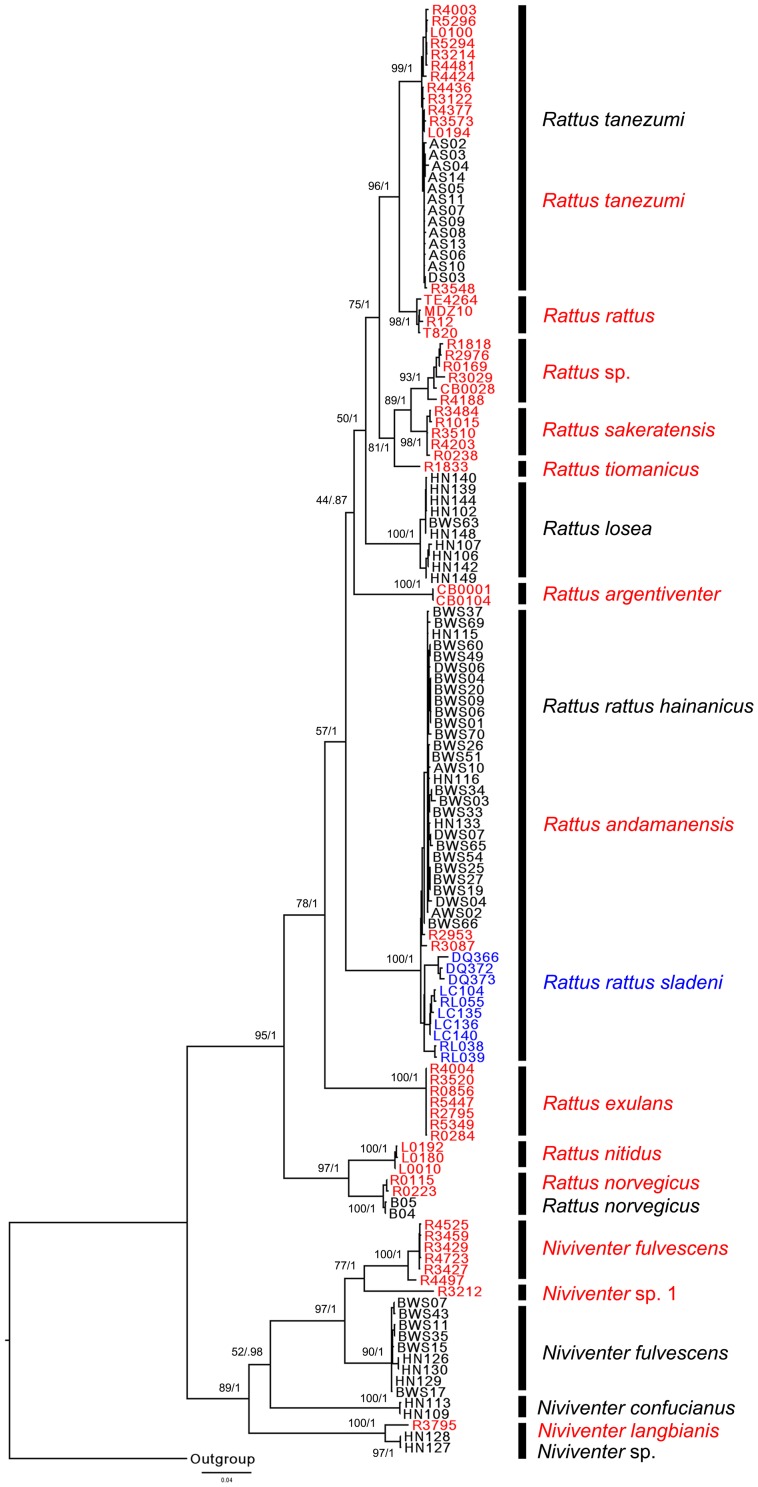
Maximum likelihood tree of *Rattus* and *Niviventer* species from Hainan, Yunnan and Southeast Asia based on the combined COI and Cyt-*b* dataset. ML and BI analyses of the dataset gave identical tree topology. Numbers beside the nodes reflect support obtained from the analysis of the dataset following two different reconstruction methods: ML/BI. The meaning of different colors of samples and lineages names is the same as in the Figure 3.

The ML tree and BI tree showed identical topology (Figure 4). According to the trees, samples of *R. norvegicus*, and *R. tanezumi* were clustered with samples of the same species collected from Southeast Asia, while samples of *R. rattus hainanicus* and *R. rattus sladeni* formed a branch with samples of *R. andamanensis*, and samples of *R. losea* formed a branch independent from *R. sakeratensis* from Southeast Asia. Samples of *N. fulvescens* and *N. confucianus* from Hainan did not cluster with any samples from Southeast Asia. Two samples of *Niviventer* sp. from Hainan showed close relationship with samples of *N. langbianis* from Southeast Asia.

**Figure 5:**
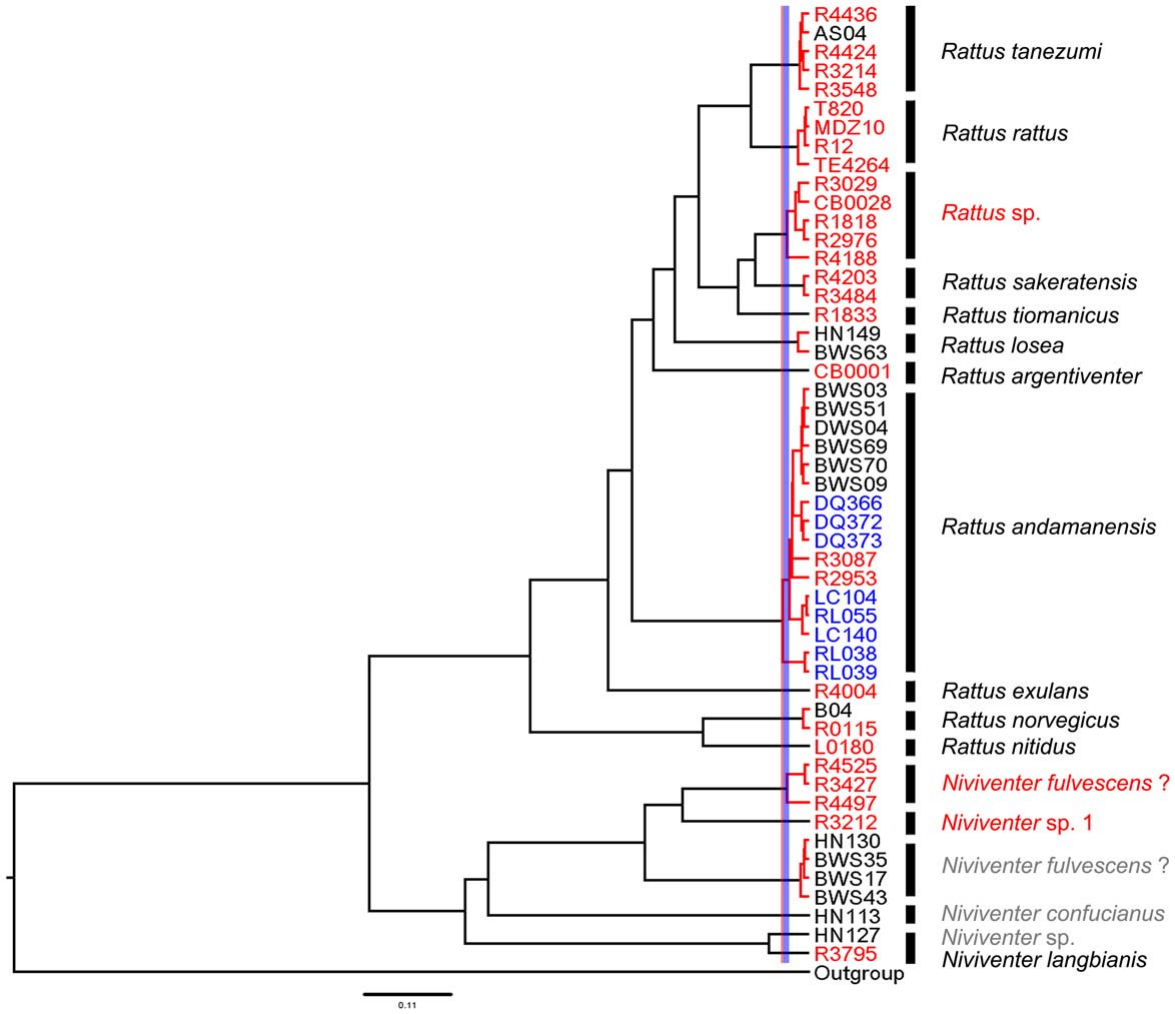
Ultrametric tree of *Rattus* and *Niviventer* species from Hainan, Yunnan and Southeast Asia based on the combined Cyt-*b* and COI dataset. Red clusters of specimens were recognized as putative species by the method of Pons et al. [19]. The blue shadow on the tree indicated the confidence interval of the threshold, and the red vertical line was the threshold point obtained from the GMYC model. The meaning of different colors of samples names is the same as in the Figure 3. The black species names were confirmed species names in published papers and this paper; the red and gray species names means they were not confirmed in published papers and this paper.

Phylogenetic based species delimitation was carried out on a series of ultrametric trees. For trees in which the NPRS method was applied, the GMYC model showed no significant fit (NJ, p = 0.1162, ML, p = 0.1756, BI, p = 0.1666). Whereas trees adjusted by PL showed significant GMYC structure in all cases, irrespective of the optimization method used (Powell, TN or Qnewt). 17 (likelihood ratio: 19.12398, p = 0.0002), 17 (likelihood ratio:13.21194, p = 0.00419) and 18 (likelihood ratio = 12.37016, p = 0.00621) species were inferred (not including the outgroup) on the NJ, ML and BI trees, respectively, including 11 and 12 species of *Rattus* and 6 species of *Niviventer*. The confidence interval for the number of species ranged from 17 to 24, which is demarcated in the blue shadow in Figure 5.

According to the species delimitation results, samples of *R. rattus hainanicus* and *R. rattus sladeni* grouped with samples of *R. andamanensis* from Southeast Asia [21] as one putative species, partly supporting the downgrading of *hainanicus* to a synonym of *R. andamanensis* by Musser and Carleton [24]. *Niviventer* specimens from Hainan were split into three putative species, and did not group with three species from Southeast Asia, even though two putative species were morphologically identified as *N. fulvescens* (Figure 5). *Niviventer* specimens collected from Kanchanaburi of Thailand, labeled as *Niviventer* sp1 [21], were related to *N. fulvenscens*, although with a molecular distance (∼0.1) outside that expected for a single species. The specimens labeled as *Niviventer* sp were placed close to *N. langbianis*, although it can not be concluded that they belong to this species, due to the position of the threshold confidence interval.

## Discussion

The distinct gap between intraspecific and interspecific variation is the cornerstone of the DNA barcoding tool for species identification [14], [15], [46]. In the present study, the range of intraspecific variation of COI for *Rattus* and *Niviventer* was low (<1.60%) while interspecific variation was high (>5.50%), almost 5–30 times higher than the average differences within species. Based on this gap, morphospecies generally formed well-supported clusters on the NJ tree. The COI data from Hainan gives 10 well-supported branches (Figure 2), meaning 10 putative species in the included samples, whereas the combined COI dataset from Hainan and Southeast Asia gave 16 well-supported groups (Figure 3). Among these branches, two *Niviventer* samples from Hainan were clustered with samples of *N. langbianis* from Laos with an average genetic distance of 1.60%, which was very small compared with the range of interspecific variation of *Niviventer* species.

In this paper, only the mitochondrial COI and Cyt-*b* genes (and no genes of the nuclear genome) were used in phylogenetic analysis and species delimitation. According to Figure 4, the phylogeny of *Rattus* species is concordant with the result in Figure 2 of Pagès et al. [21], the latter of which was based on the analysis of combined Cyt-*b*, COI and IRBP genes. Figure 4 of Pagès et al. [21], a ML tree based on Cyt-*b* and COI only, also show an identical topological relationship of *Rattus* species with Figure 2 of their paper. IRBP is a nuclear gene frequently used in phylogenetic analysis of mammals [47], [48], [49], [50]. While for the species of *Rattus*, the IRBP gene reported by Pagès et al. [21] could give only limited phylogenetic information (data not shown), thus we did not use this gene for this paper.

Species delimitation is an issue fundamental to taxonomic, evolutionary and ecological research. Use of morphological data alone in traditional species delimitation may underestimate the number of species and, in particular, may fail to identify cryptic species. Phylogenetic-based species delimitation using molecular information gives an opportunity to overcome the above weakness, hence the development of a series of analysis methods [19], [51], [52], [53]. In this paper, the GMYC method delimited 17 (NJ, ML) or 18 (BI) putative species, including 11 or 12 species of *Rattus* and 6 species of *Niviventer* from combined COI and Cyt-*b* data from Hainan and Southeast Asia (Figure 5 for the NJ tree). While the NJ tree of combined COI data (Figure 3) supported 11 species of *Rattus* and 5 species branches of *Niviventer*, which was also supported by a ML tree of combined COI and Cyt-*b* (Figure 4). According to the position of the threshold (Figure 5), *N. langbianis* (Figure 3) comprises two distinct lineages. Additionally, *R. andamanensis* (Figures 3 and 5) was split into two clusters when the BI tree was used for GMYC analysis (Data not shown). The samples of *N. langbianis* and *R. andamanensis* showed a greater range of intraspecific distance in COI than other species, with 0–1.30% for *R. andamanensis* and 0–1.60% for *N. langbianis*. According to the confidence interval of the threshold, R4188, R4497, RL038 and RL039 could be delimited as independent species (Figure 5). These samples all show a relatively large distance (0.7%–1.6%). These results suggest that where the range of intraspecific distance is great, GMYC analysis tends towards an increase in the number of species units.

The DNA barcoding results were mostly congruent with that of the species delimitation (Figure 3 and Figure 5), with 15 equivalent species assignments. The remaining clade was considered one species with the barcoding method (Figure 3), including *N. langbianis* from Southeast Asia and two samples from Hainan, but was split into two species units by the GMYC model (Figure 5), because of the relative high genetic distance between them. The congruent results add weight to the 15 molecular species assignments, and suggest the presence of a strong signal in the molecular data of the groups researched here. On the other hand, the diversity of *Rattus* and *Niviventer* species of China and Southeast Asia revealed by molecular data indicate that further taxonomic study is required for these two genera.

The combined results of morphological identification, DNA barcoding and molecular species delimitation showed that there are four *Rattus* species (*R. tanezumi, R. norvegicus, R. losea, and R. andamanensis*), three *Niviventer* species, and *Callosciurus erythraeus*, *Neohylomys hainanensis*, *Tupaia belangeri* and *Suncus murinus*, in the investigated area of Hainan (especially in and nearby residential environments). According to the collection records, almost all specimens of *R. tanezumi* and *R. norvegicus* were collected indoors, while *R. losea* and *R*. *andamanensis* were all trapped in farmlands and forests around residential sites. In the laboratory, collected blood samples of small mammals were checked to determine whether they were infected by Richettsiae bacteria. Only *N. fulvescens* from Chengmai county were found to harbor the bacteria, with over half of the specimens PCR positive (seven), and seven isolates obtained [22]. The high infection rate of *N. fulvescens* in Hainan and another report [54] indicate that this species is an important host animal of *Rickettsia* bacteria in Southern China. The accurate identification of small mammals can give information on, not only the host animal of specific pathogens, but also the possible distribution of related diseases according to the distribution of host animals, which is very important in zoonotic disease control and prevention.

There are four subspecies of *R. rattus* recorded in China: *R. rattus rattus*, *R. rattus alexandrinus*, *R. rattus sladeni* and *R. rattus hainanicus*. The last two subspecies were only recorded in China, and all are wild species as opposed to commensal rodents [23]. In contrast, Musser and Carleton [24] regarded *sladeni* as a synonym of *R. tanezumi*, and *hainanicus* as a synonym of *R. andamanensis*. The results from this study confirmed that samples of *R. rattus hainanicus* from Hainan Island, *R. rattus sladeni* of Yunnan and *R. andamanensis* from Southeast Asia belong to one species. The genetic distance in COI of these samples ranged from 0 to 1.3%, with an average value of 0.34%. The preferred habitation of *R. rattus hainanicus* recorded in the investigation was also similar to that of *R. andamanensis* (*R. sikkimensis* in Aplin et al. [25]).

*Rattus losea* was described from Taiwan, with morphologically identical or similar specimens recorded from East and Southeast Asia. However, Aplin et al. [25] reported that *R. losea* was discontinuously distributed across mainland Southeast Asia and East Asia, and that morphological variation existed between two geographic populations. Accordingly, Pagès et al. [21] did not name samples collected only from Southeast Asia as *R. losea* although these samples possessed the morphological characters of this species and formed a single independent group. Aplin et al. [27] confirmed that the *losea*-like rats of Southeast Asia should be named *R. sakeratensis*. Our research further confirmed the caution of Pagès et al. [21] and the result of Aplin et al. [27] with the samples from Hainan.

The *Niviventer* genus is a diverse group distributed throughout East and Southeast Asia, with nine individual species recorded in China to date [31], [55]. Among them, Wang [55] reported that *N. confucianus lotipes* and *N. fulvescens* had been recorded in Hainan. In the list of Musser and Carleton [24], *N. tenaster* and *N. fulvescens* occurred on Hainan Island, since these authors regarded *lotipes* as a synonyms of *N. tenaster*.

The NJ tree of COI in our study demonstrates that there are three independent lineages of *Niviventer* collected in Hainan (Figure 2). After checking the morphological characteristics of the three voucher specimens of each putative species, the two clades could be named as *N. confucianus* (HN113) and *N. fulvescens* (HN162). Whereas the voucher specimen (HN131) for the third clade was a white-bellied rat with a mono-colored dark brown tail. There are only two known *Niviventer* species with mono-colored dark tails, *N. cremoriventer* and *N. langbianis* [28]. *N. cremoriventer* is recorded in Yunnan of China [31], [55], while *N. langbianis* has no record in China, but likely to be found in Southern China according to its distribution in Southeast Asia [28]. The voucher specimen has a relatively large bullae and long anterior incisive foramina, and could be identified as *N. langbianis*. The DNA-based species delimitation gave more complex results than that of the COI NJ tree. The *N. fulvescens* from Hainan and Southeast Asia formed two independent clades. We could not confirm which one was the true *fulvescens* although it was also discussed by Pagès et al. [21]. As with the *N. langbianis*-like individual from Hainan and Southeast Asia, the *langbianis*-like specimens from Hainan should not be named until further studies of morphology, ecology and genetics are carried out. For the third *N. confucianus*-like species found in Hainan, sufficient molecular characters of *N. tenaster* were necessary to confirm whether it was *N. confucianus* or *N. tenaster*. However, a series of works were carried out using only the Cyt-*b* gene, in order to explore the species level phylogenetics and phylogeography of members from China and Vietnam [31], [56], [57], [58]. Using these sequences there was insufficient information (data not shown) supporting whether the *confucianus*-like specimens found in Hainan were *N. tenaster*. Accordingly, more molecular data and increased sampling are necessary to confirm the systematic position of these individuals from Hainan.
